# Beneficial Effect of Traditional Chinese Medicinal Formula Danggui-Shaoyao-San on Advanced Glycation End-Product-Mediated Renal Injury in Streptozotocin-Diabetic Rats

**DOI:** 10.1155/2012/140103

**Published:** 2011-08-07

**Authors:** I-Min Liu, Thing-Fong Tzeng, Shorong-Shii Liou, Chia Ju Chang

**Affiliations:** ^1^Department of Pharmacy and The Graduate Institute of Pharmaceutical Technology, Tajen University, Yanpu Shiang, Ping Tung Shien 90701, Taiwan; ^2^Department of Internal Medicine, Pao Chien Hospital, Ping Tung City 90065, Taiwan; ^3^School of Chinese Pharmaceutical Sciences and Chinese Medicine Resources, China Medical University, Taichung 40402, Taiwan

## Abstract

The present study was undertaken to characterize the effects of Danggui-Shaoyao-San (DSS), a famous traditional Chinese medicine formula consisting of six herbal medicines, on diabetic nephropathy. Streptozotocin-induced diabetic rats were orally administrated DSS (2.8 g kg^−1^ per day) for 12 consecutive weeks. DSS partially decreased the high plasma glucose level in diabetic rats. Diabetic-dependent alterations in urinary albumin, 24-hour urinary albumin excretion rate, and creatinine clearance as well as the kidney hypertrophy (kidney weight/body weight ratio) and glomerular mesangial matrix expansion were ameliorated after 12 weeks of DSS treatment. The increased expression of nuclear factor-**κ**B as well as transforming growth factor-**β**_1_ and the progressive accumulation of type IV collagen in kidney of diabetic rats were also attenuated by DSS. Not only the elevated levels of advanced glycation end products (AGEs) and *N* ^**ε**^-(carboxymethyl)lysine but also the higher levels of lipid peroxidation products in kidney of diabetic rats were ameliorated by DSS. Decreased activity of superoxide diamutase and glutathione peroxidase in kidney of diabetic rats was enhanced by DSS. These data demonstrated that the renoprotective effects of DSS in STZ-diabetic rats not only were attributable to regulate plasma glucose to attenuate AGEs expression in diabetic glomeruli but also likely reflected its antioxidant activity.

## 1. Introduction

A dramatic worldwide increase in the number of patients with diabetes has been reported, and the disease is becoming a serious social problem. Among diabetic complications, nephropathy is the most common cause of end-stage renal disease (ESRD) in developed countries and a major cause of morbidity and mortality in patients with diabetes [1]. It is characterized by structural abnormalities including hypertrophy of both glomerular and tubular elements, increase in the thickness of glomerular basement membranes, and progressive accumulation of extracellular matrix components [2]. It also results in functional alterations including the early increase in the glomerular filtration rate with intraglomerular hypertension, subsequent proteinuria, systemic hypertension, and eventual loss of renal function [2]. The development of irreversible renal change in diabetes mellitus such as glomerulosclerosis and tubulointerstitial fibrosis results ultimately in ESRD [1]. Measures to prevent the appearance and progression of diabetic nephropathy should therefore be instituted as early as possible. Although adequate control of blood glucose levels may prevent the development of complications, it is difficult to achieve strict blood glucose control, leading to a year-by-year increase in the number of patients with diabetes [3]. 

Beyond glycaemic control, other metabolic factors have been shown to be involved in the development of diabetic kidney disease, that is, advanced glycation end products (AGEs). Furthermore, an adequate control of high blood pressure and treatment of microalbuminuria are major therapeutic targets. To achieve adequate blood pressure control, a combination therapy with different classes of antihypertensive agents is often necessary, especially including angiotensin-converting enzyme inhibitors (ACEIs) and angiotensin receptor blockers (ARBs) [4]. Besides hyperglycaemia and high blood pressure, other risk factors have been identified in the development or progression of diabetic kidney disease, such as hyperlipidaemia and obesity. The increased lipid peroxidation in the kidney implies the level of susceptibility to diabetic oxidative stress, leading to diabetic complications. From this view point, prevention of hyperlipidemia and/or lipid peroxidation resulting from oxidative stress is considered to play a crucial role in protection from disorders associated with diabetes [5]. Therefore, new drugs for treatment of diabetic nephropathy based on different mechanisms of action are needed.

Danggui-Shaoyao-San (DSS), also called Toki-shakuyaku-san or TJ-23, comprising Radix *Paeoniae Alba*, Radix *Angelica sinensis*, Rhizoma *Chuanxiong*, *Poria cocos*, Rhizoma *Atractylodis macrocephalae*, and Rhizoma *Alismatis*, is a widely used formula of traditional Chinese medicine (TCM) derived from “Jingui Yaolue”, a medical classic written by Zhongjing Zhang in the Eastern Han Dynasty. Identification and determination of the major constituents in traditional Chinese medicinal prescriptions is constantly being carried. Researchers have much information on a lot of active fractions and components from DSS [6]. Monoterpene glycosides, phenolic compounds, and phthalides are the most representative components of DSS as far as both the contents and their biological activities are concerned. Monoterpene glycosides are responsible for the efficacy of R. *Paeoniae Alba*. A case in point is albiflorin and paeoniflorin, which exhibits analgesia, spasmolysis, anti-inflammation, and anticoagulation activities [7]. Phenolic acids and phthalides in R. *Angelica sinensis *and R. *Chuanxiong *also have vasodilatative, antithrombotic, antioxidative, anti-inflammatory, and muscle relaxant effects [8]. In addition, atractylenolides from R. *Atractylodis macrocephalae* showed gastrointestinal inhibitory, anti-inflammatory, and antioxidative activity [9]. Meanwhile, cytotoxic, anti-inflammatory, and antioxidant activity of triterpenes in R. *Alismatis *and *Poria cocos *has also been documented [10, 11]. DSS has been used in China as a blood-activating and stasis-eliminating drug to treat gynecological disorders such as dysmenorrhea, amenorrhea, and infertility without observation of side effects for thousands of years [12, 13]. Recent studies show that it also possesses the capability of treating neural dysfunctions such as senile dementia, memory loss, and other cognitive disorders; thus the formula is used as a remedy for Alzheimer's disease in Japan [14, 15]. The amelioration of streptozotocin diabetes-induced renal damage by the herbal formula containing *Poria cocos*, R. *Alismatis*, and R. *Atractylodis macrocephalae* has been documented [16]. Although so many beneficial effects have been shown, the possibility of DSS is beneficial in the treatment of diabetic nephropathy has not been previously explored. 

Diabetes usually can be classified as type 1 or type 2 on the basis of the patient's clinical presentation. It is often stated that type 1 diabetes results from a complex interplay between varying degrees of genetic susceptibility and environmental factors. Patients with diabetes mellitus type 1 present with an extensive risk for microvascular complications like retinopathy, nephropathy, and peripheral neuropathy. The natural history of diabetic nephropathy differs according to the type of diabetes and whether microalbuminuria is present. If untreated, 80% of people who have type 1 diabetes and microalbuminuria will progress to overt nephropathy, whereas only 20–40% of those with type 2 diabetes over a period of 15 years will progress [17]. The present study was conducted to characterize the efficacy of DSS on diabetic nephropathy of type 1 diabetes utilizing streptozotocin-induced diabetic rats (STZ-diabetic rats) as an animal model.

## 2. Methods

### 2.1. Materials

The concentrated powders for DSS (Cat., no. 3520), made of 100% pure authentic Chinese herbs of highest qualities, were produced by Kaiser Pharmaceutical Co., Ltd. (Tainan, Taiwan) under internationally certified Good Manufacturing Practices guidelines. The experienced botanists and chemists in the supplier use macroscopic and microscopic examinations as well as thin-layer chromatography and high-performance liquid chromatography identification to authenticate the plants, plant parts used, and processed raw herbs. The reference specimens were deposited at the herbarium of supplier to permit future reference and verification. DSS is composed of the following 6 raw materials in the dry weight ratio of 3 : 16 : 8 : 4 : 4 : 8. *Angelica sinensis *(Oliv.) Diels (family: Umbelliferae), *Paeonia lactiflora *Pall. (family: Ranunculaceae), *Ligusticum chuanxiong* Hort. (family: Umbelliferae), *Poria cocos* (Schw.) Wolf (family: Polyporaceae), *Atractylodes macrocephala* Koidz. (family: Compositae), and *Alisma orientalis* (Sam.) Juzep. (family: Alismaceae). The formula is cooked as a soup, combining all ingredients at the appropriate amount of distilled water. Low temperature processing is employed to slowly concentrate the extracts, protecting, releasing, and maintaining all essential ingredients. Aqueous extract of total ingredients was concentrated to 3 g of dry weight to meet the ratio of 5 : 1 in the powder of DSS. Streptozotocin (STZ) and sodium pentobarbital were purchased from Sigma-Aldrich, Inc. (Saint Louis, Mo, USA). The diagnostic kits for determinations of glucose (Cat., no. COD12503), cholesterol (Cat., no.COD11539), and triglyceride (Cat., no. COD11529) in plasma were purchased from BioSystem (Barcelona, Spain). Nephrat II enzyme-linked immunosorbent assay (ELISA) kit (Cat., no. NR002) was obtained from Exocell, INC. (PA, PUA). The diagnostic kits for determinations of creatinine concentration in serum or urine (Cat., no. 221-30), and kinetic reagent for measurement of blood urea nitrogen (BUN) (Cat., no. 283-30) were purchased from Diagnostic Chemicals Limited (Conn, USA). Rabbit polyclonal antinuclear factor (NF)-*κ*B p65 antibody (Cat., no. sc-109) was purchased from Santa Cruz Biotechnology, Inc. (Santa Cruz, Calif, USA). Rabbit polyclonal antitransforming growth factor (TGF)-*β*_1_ antibody (Cat., no. CA0290) was purchased from Cell Applications, Inc. (Calif, USA). Rabbit polyclonal antitype IV collagen antibody (Cat., no. ab6586) was from Abcam plc (Cambridge, UK). Labeled streptavidin biotin reagent and liquid diaminobenzidine (DAB)+ substrate chromogen system were purchased from DAKO Corporation (Calif, USA). Anti-*N* ^*ε*^-(carboxymethyl)lysine (CML) rat autoantibody ELISA kit (Cat., no. CY-8069) was from MBL International (Mass, USA). Bioxytech LPO-586 kit was obtained from OXIS International (Portland, USA). The colorimetric assay kits for measurements of superoxide dismutase (SOD, Cat., no. 706002), and glutathione peroxidase (GSH-Px, Cat., no. 703102) activities in plasma were purchased from Cayman Chemical (Mich, USA). The protein assay kit was obtained from Bio-Rad Laboratories (Calif, USA). All analyses were performed in accordance with the manuals provided by the manufacturers.

### 2.2. Animal Models

Male Wistar rats, aged 8–10 weeks, were obtained from the National Laboratory Animal Center (Taipei, Taiwan). They were maintained in a temperature-controlled room (25 ± 1°C) and kept on a 12 : 12 light-dark cycle (light on at 06:00 h) in our animal center. Food and water were available *ad libitum*. STZ-diabetic rats were prepared by intravenously (i.v.) injecting 60 mg/kg STZ into male Wistar rats. Animals were considered to be diabetic if they had plasma glucose concentrations of 350 mg/dL or greater in addition to polyuria and other diabetic features. All studies were carried out 2 weeks after the injection of STZ. All animal procedures were performed according to the Guide for the Care and Use of Laboratory Animals of the National Institutes of Health as well as the guidelines of the Animal Welfare Act.

### 2.3. Treatment Protocols

One-gram powders of DSS were well dispersed in 5 mL of distilled water to have the 100% solution for treatment. A metabolism coefficient of 6.25 was employed to convert the recommended daily dosage of DSS at 27 g for adult into rats, assuming that average body weight of an adult is 60 kg [18]. Thus, STZ-diabetic rats daily received oral treatment with DSS at 2.8 g kg^−1^. Another group of STZ-diabetic rats and nondiabetic rats received the equivalent volume of distilled water used to prepare the preparation. All animals were administered once daily via gastric tube. The standard rat diet and water were available *ad libitum* throughout the entire treatment period. Every four weeks after the treatment, rats were weighed, and blood samples were collected from a tail vein; meanwhile, individual rat was placed in metabolic cages (Shineteh Instruments Co., Ltd., Taipei, Taiwan) to obtain 24-hour urine collections for measurements of urine creatinine (Cr) and albumin concentrations. The systolic blood pressure (SBP) of the tail artery was also measured at monthly intervals.

Rats were sacrificed at the end of the 12-week treatment when the rats were deeply anaesthetized by sodium pentobarbital. Kidneys were dissected and rinsed with cold isotonic saline and then weighed. An index of renal hypertrophy was estimated by comparing the wet weight of the left kidney to the body weight. Thereafter, and some kidney tissues were put at once in liquid nitrogen and stored at −80°C for biochemical and enzymatic determinations, some were fixed in 10% neutralized formalin for histology and immunohistochemistry.

### 2.4. Blood Sampling and Analysis

Blood sample of rats were centrifuged at 2,000 g for 10 minutes at 4°C, and plasma was removed and aliquot for the respective analytical determinations. The diagnostic kits for determinations for plasma levels of glucose (Cat., no. COD12503), cholesterol (Cat., no. COD11539), and triglyceride (Cat., no. COD11529) were purchased from BioSystem (Barcelona, Spain). The serum creatinine (Cr) concentration was determined by the commercial assay kit (Cat., no. 221-30) purchased from Diagnostic Chemicals Limited (Conn, USA). Blood urea nitrogen (BUN) was determined by kinetic reagent (Diagnostic Chemicals Limited, Cat., no. 283-30). All analyses were performed in accordance with the manuals provided by the manufacturers. 

### 2.5. Analysis of Urine Parameters

The 24-h urine collected from each diabetic rat and age-matched control was centrifuged at 2,000 g for 10 min. Urinary albumin concentrations were measured by Nephrat II enzyme-linked immunosorbent assay (ELISA) kit (Cat., no. NR002) obtained from Exocell, INC. (PA, PUA). The concentration of Cr in pooled urine samples was determined by the commercial assay kit (Diagnostic Chemicals Limited, Cat., no. 221-30). All analyses were performed in accordance with the manuals provided by the manufacturers. The 24-h urinary albumin excretion rate (UAER) was calculated as: UAER (*μ*g 24 h^−1^) = urinary albumin (*μ*g mL^−1^) × 24-h urine volume (mL). Cr clearance (Ccr) was calculated using the following equation: Ccr (mL min^−1^ kg^−1^) = [urinary Cr (mg dL^−1^) × urinary volume (mL)/serum Cr (mg dL^−1^)] ×  [1000/body weight (g)] × [1/1440 (min)].

### 2.6. Blood Pressure Measurement

The SBP of the tail artery was measured by noninvasive blood pressure system (MODEL BP-6, Diagnostic & Research Instruments Co., Ltd., Taoyuan, Taiwan). The measurements for SBP were recorded in quadruplicate for each rat, and the average blood pressure was calculated.

### 2.7. Renal Histological Analysis

 For morphometric analysis, the kidney was removed and embedded in paraffin to prepare 4-*μ*m tissue slices. The tissue slices were stained with periodic acid-Schiff (PAS). The mesangial expansion index (MEI) was scored in four levels from 0 to 3, with the index scores defined as follows [19]: 0, normal glomeruli; 1, matrix expansion occurred in up to 50% of a glomerulus; 2, matrix expansion occurred in 50 to 75% of a glomerulus; 3, matrix expansion occurred in 75 to 100% of a glomerulus. Scores were assigned for at least 30 glomeruli from kidney slices from each animal, and the means were calculated. Each slide was scored by a pathologist who was unaware of the experimental details.

### 2.8. Immunohistochemistry Staining

 For immunohistochemical staining, renal tissues were fixed in 10% neutral buffered formalin, casted in paraffin, sliced into 4 *μ*m sections, and placed onto microscope slides. After removal of the paraffin by xylene and dehydration by graded alcohol, slides were immersed into distilled water. Kidney sections were then transferred into a 10 mmol L^−1^ citrate buffer solution and heated at 80°C for 5 minutes for antigen retrieval. After washing, 3.0% peroxide was applied for 20 minutes to block the activity of endogenous peroxidase. To avoid nonspecific staining, slides were incubated with normal goat serum at room temperature for 20 minutes. The sections were then incubated in blocking solution (5% BSA) for 1 hour at room temperature, followed by treatment with anti-NF-*κ*B p65 antibody (1 : 200), anti-TGF-*β*_1_ antibody (1 : 200), or antitype IV collagen antibody (1 : 500), where indicated overnight at 4°C. Negative control sections were stained under the identical conditions by substituting the primary antibody with equivalent concentrations of normal rabbit IgG. After washing with phosphate buffered saline, the slides were incubated with the labeled streptavidin biotin reagent, following the manufacturer's instructions. Immunoreactive products were made visible by DAB reaction. Sections were counterstained with haematoxylin for 15 seconds. Brownish yellow granular or linear deposits were interpreted as positive areas. To evaluate the immunostaining, a total of more than 30 randomly chosen glomeruli per rat was coded and graded in a blind manner. Each score reflects changes in the extent rather than the intensity of staining and depends on the percentage of positive glomeruli. The degree of TGF-*β*_1_ and type IV collagen expression in four rats from each group was graded as follows: 0, absent or less than 25% staining; 1, 25–50% positive staining; 2, 50–75% positive staining; 3, more than 75% positive staining [20].

### 2.9. Renal Advanced Glycation End Products (AGEs) Level

The renal AGE level was determined according to previous method with slight modifications [21]. Minced kidney tissue was delipidated with chloroform and methanol (2 : 1, v/v) overnight. After washing, the tissue was homogenized in 0.1 N NaOH, followed by centrifugation at 8,000 g for 15 min at 4°C. The amounts of AGEs in these alkali-soluble samples were determined by measuring the fluorescence at an emission wavelength of 440 nm and an excitation wavelength of 370 nm using a fluorescence spectrophotometer (F-4500, Hitachi, Japan). A native bovine serum albumin (BSA) preparation (1 mg mL^−1^ of 0.1 N NaOH) was used as a standard, and its fluorescence intensity was defined as one unit of fluorescence. The fluorescence values of samples were measured at a protein concentration of 1 mg mL^−1^ and expressed in AU compared with a native BSA preparation.

### 2.10. Assessment of Renal *N* ^*ε*^-(carboxymethyl)lysine (CML)

The kidney was homogenized in ice-cold buffer (0.1 mmol L^−1^ KH_2_PO_4_/K_2_HPO_4_, pH 7.0, plus 29.2 mg ethylenediaminetetraacetic acid in 100 mL of distilled water and 10 mg digitonin in 100 mL of distilled water, final volume, 2,000 mL) to produce a homogenate. The kidney homogenates were then centrifuged at 10,000 g for 10 min at 4°C. Then, the supernatant was tested for CML using the anti-CML rat autoantibody ELISA kit which employs the semiquantitative enzyme immunoassay technique. The absorbance of the resulting yellow product is measured at 450 nm. 

### 2.11. Estimation of Renal Lipid Peroxidation Products

Kidney was homogenated as described above and centrifuged at 10,000 g for 10 min at 4°C. The supernatant was collected and immediately tested for lipid peroxidation products using the Bioxytech LPO-586 kit. The kit uses a chromatogenic reagent which reacts with the lipid peroxidation products malondialdehyde and 4-hydroxyalkenals at 45 ± 1°C, yielding a stable chromophore with maximum absorbance 586 nm.

### 2.12. Measurement of Renal Antioxidant Enzyme Activity

Total SOD (E.C.: 1.15.1.1) activity evaluated in kidney homogenates was determined by commercial kit for measuring its ability to inhibit the photochemical reduction of tetrazolium salt in absorbance at 450 nm. The activity assay for GSH-Px (E.C.: 1.11.1.9) determined by commercial kit was based on the oxidation of NADPH to NAD+, catalyzed by a limiting concentration of glutathione reductase, with maximum absorbance at 340 nm. Data are expressed as units (U) per mg of protein as compared with the standard. The total protein in each tissue lysate was measured using Bio-Rad protein assay kit.

### 2.13. Statistical Analysis

Data are expressed as the mean ± SD for each group of animals at the number (*n*) indicated in tables. Statistical differences among groups were determined by using two-way repeated measures ANOVA. The Dunnett range *post hoc *comparisons were used to determine the source of significant differences where appropriate. A *P* value <0.05 was considered statistically significant.

## 3. Results

### 3.1. General Characteristics of Rats

At the beginning of the treatment, the plasma glucose was significantly increased in vehicle-treated STZ-diabetic rats as compared to nondiabetic group; these changes were more marked at the 12th week following diabetes induction (Table 1). Although the plasma glucose in STZ-diabetic rats receiving DSS treatment for 4 weeks was somewhat lower than the corresponding values for vehicle-treated group, these differences did not achieve statistical significance (Table 1). The plasma glucose lowering effect was more markedly when STZ-diabetic rats receiving DSS treatment for 12 weeks (Table 1). The DSS-induced plasma glucose lowering effect in STZ-diabetic rats was persisted to the end of the treatment. DSS also abridged the weight gain loss in diabetic rats at the end of the experimental period (Table 1).

In addition, the plasma levels of cholesterol and triglyceride were markedly higher in vehicle-treated STZ-diabetic rats than those in nondiabetic group (Table 1). Plasma levels of cholesterol and triglyceride in STZ-diabetic rats were slightly lower by DSS at all time points throughout the study (Table 1). However, DSS made no influence on SBP in STZ-diabetic rats during entire experiments (Table 1).

### 3.2. Changes in Renal Function-Related Parameters

During the study period, levels of serum Cr and BUN in STZ-diabetic rats were higher than those of nondiabetic group (Table 2). At the termination of 12 weeks of DSS treatment, the values for serum Cr and BUN of STZ-diabetic rats were lower than those of their vehicle-treated counterparts (Table 2). The urine volume in STZ-diabetic rats was markedly (*P* < 0.01) greater than that in nondiabetic rats throughout the experimental period (Table 2). Lowering urine volume in diabetic rats received DSS treatment was exhibited at all time points throughout the experiments (Table 2). Actually, the urine volume in DSS-treated STZ-diabetic rats was still higher than the value from nondiabetic rats at any time point throughout the study (Table 2).

The levels of Ccr and UAER in diabetic rats were markedly (*P* < 0.05) increased as compared with those for nondiabetic rats; these values in STZ-diabetic rats were kept increasing as relative to that in nondiabetic group at the end of the study (Table 2). Treatment of STZ-diabetic rats with DSS resulted in a partial reversal of these abnormal parameters, and the effects of DSS were persisted at the end of 12-week treatment (Table 2).

### 3.3. Influences on the Kidney Hypertrophy

At the end of study period, the mean kidney weight and the ratio of kidney weight to body weight in vehicle-treated STZ-diabetic were significantly increased as compared to those in the nondiabetic group (*P* < 0.01, Table 3). Treatment STZ-diabetic rats with 12 weeks of DSS reduced the degree of renal hypertrophy (Table 3).

### 3.4. Influence on the Renal Histology

**Figure 1:**
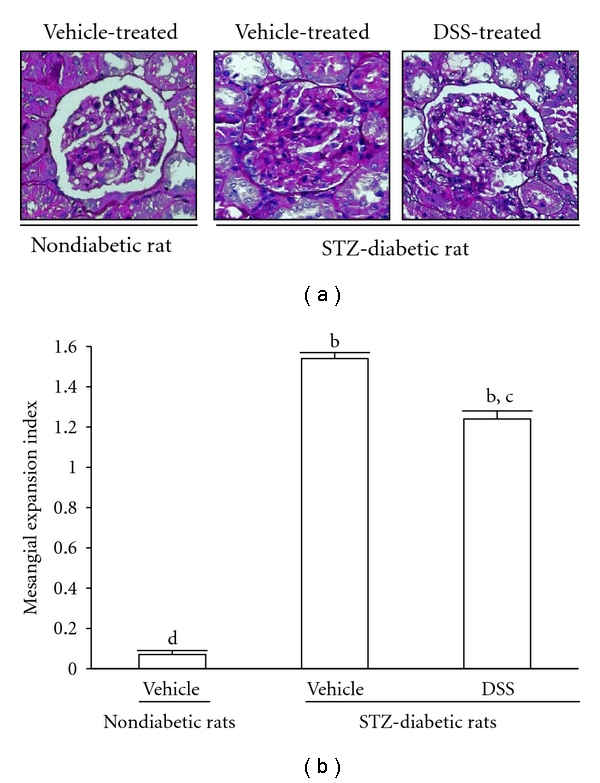
(a) Representative photomicrographs (original magnification, 200x) of PAS-stained kidney sections from STZ-diabetic rats receiving 12 weeks of DSS (2.8 g/kg/day) treatment. The vehicle (distilled water) used to disperse DSS was given at the same volume. (b) Expansion of the glomerular matrix was scored using 4 levels, and an average value was obtained from analyses of more than 30 glomeruli per rat. Values (mean ± SD) were obtained for each group of 4 animals. ^b^ *P* < 0.01 compared to the values of vehicle-treated nondiabetic rats. ^c^ *P* < 0.05 and ^d^ *P* < 0.01 compared to the values of vehicle-treated STZ-diabetic rats, respectively.

Mesangial matrix fraction of glomeruli observed in PAS-stained images has been shown in Figure 1(a). Although the effect induced by DSS was not achieved to that from normal rats, mesangial matrix fraction in diabetic rats received a 12-week treatment with DSS was lower than that of their vehicle-treated counterparts. The index of the glomerular matrix expansion in each study group was shown in Figure 1(b).

### 3.5. Influence on the Renal Expression of NF-*κ*B, TGF-*β*_1_, and Type IV Collagen

**Figure 2:**
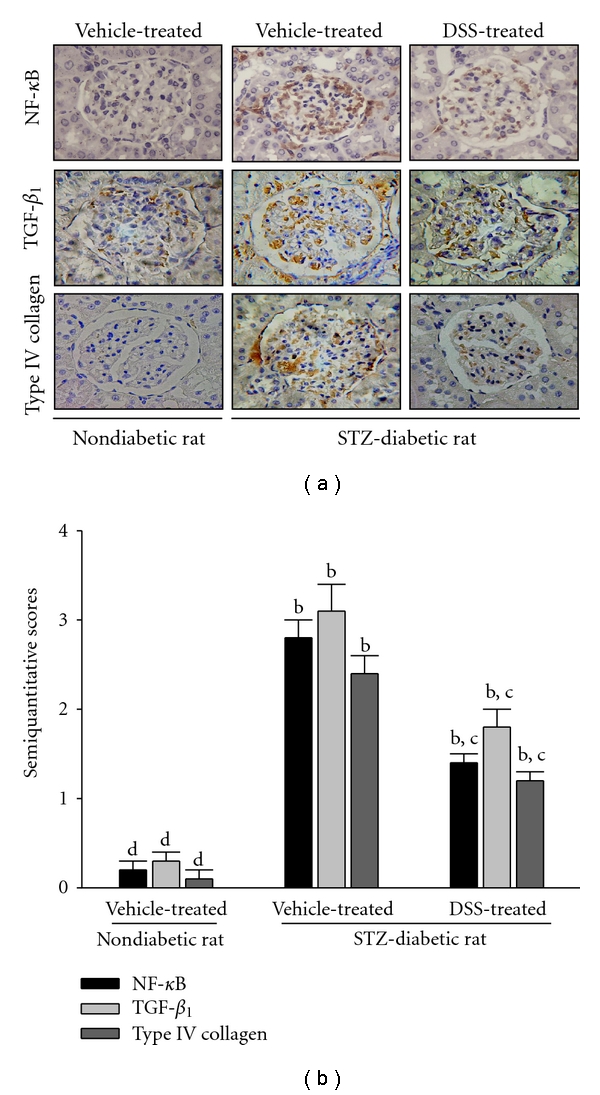
(a) Renal immunostaining for NF-*κ*B, TGF-*β*_1_, and type IV collagen expression in STZ-diabetic rats receiving 12 weeks of DSS (2.8 g kg^−1^ per day) treatment. Original magnification, 200x. The vehicle (distilled water) used to disperse DSS was given at the same volume. (b) Semiquantitative assessments of the immunostaining for proteins were scored using 4 levels, and an average value was obtained from analyses of more than 30 glomeruli per rat. Values (mean ± SD) were obtained for each group of 4 animals. ^b^ *P* < 0.01 compared to the values of vehicle-treated nondiabetic rats, respectively. ^c^ *P* < 0.05 and ^d^ *P* < 0.01 compared to the values of vehicle-treated STZ-diabetic rats, respectively.

Renal immunostaining for NF-*κ*B expression in STZ-diabetic rats receiving 12 weeks of DSS (2.8 g kg^−1^ per day) treatment has been indicated in Figure 2(a). Semi-quantitative assessment of the immunostaining for the protein indicating that the NF-*κ*B expression in vehicle-treated STZ-diabetic rats was markedly higher than that of vehicle-treated nondiabetic rats (Figure 2(b)). The elevated NF-*κ*B protein expressions in kidney of STZ-diabetic rats were reduced by 12 weeks of DSS treatment (Figure 2(a)). The STZ-diabetic rats exhibited greater TGF-*β*_1_ immunostaining in glomeruli; treatment with DSS at the 12th week showed reduced TGF-*β*_1_ protein in glomeruli (Figure 2). The type IV collagen was accumulated in the glomeruli of STZ-diabetic rats relative to that of nondiabetic group. After 12 weeks of experimental period, the extents of type IV collagen expression in glomeruli of DSS-treated STZ-diabetic rats were clearly less than that of their vehicle-treated counterparts (Figure 2). 

### 3.6. Influence on the Renal AGEs, CML, and Lipid Peroxidation Products

Both the higher renal levels of AGEs and CML in vehicle-treated STZ-diabetic rats were effectively lowered by 12 weeks of 2.8 g kg^−1^ per day DSS treatment (Table 4). Meanwhile, the lipid peroxidation products level was significantly elevated in STZ-diabetic rats. At the termination of 12| weeks of DSS treatment, the increased renal lipid peroxidation products in STZ-diabetic rats were significantly reduced as relative to their vehicle-treated counterparts (Table 4).

### 3.7. Changes on Antioxidant Enzyme Activity in Renal Cortex

Although significant decrease in SOD and GSH-Px activity in kidney was found in STZ-diabetic rats as compared with those of the nondiabetic group, both the activities of SOD and GSH-Px in kidney of DSS-treated STZ-diabetic rats were significantly higher than values in vehicle-treated group at the end of the 12-week treatment (Table 4).

## 4. Discussion

Diabetic nephropathy is a progressive disease that causes glomerular fibrosis and impairment of renal function, with progression over time. Urinary albumin excretion has been demonstrated to be a good clinical predictor of renal lesions in diabetic nephropathy [22, 23]. Increase in urinary albumin concentration corresponding to the hyperglycemia was observed in rats following diabetes induction. Furthermore, serum levels of creatinine and urea nitrogen as well as creatinine clearance, which are generally considered as markers of renal function, were higher in STZ-diabetic rats than those of nondiabetic group, implying the presence of diabetic kidney disease with renal hyperfiltration. We observed that repeated treatment with DSS could attenuate albuminuria as well as ameliorate the loss of renal function and glomerular hyperfiltration in STZ-diabetic rats. In addition to the increase in urinary albumin excretion, one of the most remarkable renal pathological findings in diabetic nephropathy is mesangial expansion due to pathological accumulation of extracellular matrix (ECM) components in glomeruli [24]. Considering that type IV collagen is the major constituent in the basement membranes of kidney [25], immunohistological staining of type IV collagen in glomeruli was performed to further clarify the effect of DSS on glomerular fibrosis. In our experimental diabetic rat model, type IV collagen was strikingly accumulated in the mesangial area of glomeruli but was expressed at lower levels in glomeruli of STZ-diabetic rats treated with 12 weeks of DSS. Furthermore, the accelerated mesangial expansion in glomeruli of STZ-diabetic rats was remissive after DSS treatment. The renal histological analysis reflected that this prescription has potential effects on the deterioration of renal fibrosis through reduction of renal ECM accumulation in STZ-diabetic rats. 

Much attention has been focused on exploring mechanisms related to the development of diabetic nephropathy. At present, several growth factors have been proposed to be involved in mediating the development of diabetic renal hypertrophy; among them, the multifunctional cytokine TGF-*β*_1_ is known to be upregulated in diabetic kidney [26]. TGF-*β*_1_ is believed to contribute to a prominent role in the mesangial cell proliferation and ECM production, the major pathological changes in early diabetic nephropathy [26]. Thus, TGF-*β*_1_ has been considered as a therapeutic target in fibrotic disease such as diabetic nephropathy and other chronic kidney diseases [27]. It was worth noting that the overexpression of TGF-*β*_1_ in glomeruli of diabetic rats was lessened following the lowering of plasma glucose underlying DSS treatment. The plasma glucose lowering effect induced by treatment of diabetic rats with DSS represents an obvious factor in the assessment of the mechanism(s) by which this formula may have prevented renal functional and structural changes in the diabetic rats. 

It has long been known that hypertension is an aggravating factor in increased intraglomerular pressure and may be the key hemodynamic determinant of diabetic renal injury as well; it is therefore well recognized that blood pressure control is important in diabetic patients [5]. Actually, DSS was observed to make less influence on the blood pressure in STZ-diabetic rats throughout the study period. These data have implications that DSS exhibits a renoprotective effect in an animal model of diabetic nephropathy by inhibiting the expression of TGF-*β*_1_, which might be linked to its beneficial effect on the hyperglycemic state but did not correlate with blocking intrarenal renin angiotensin system. 

The hyperglycemia condition, a chronic metabolic disorder of glucose, results in irreversible tissue damage by the protein glycation reaction, which leads to the formations of glycosylated protein and AGEs [2]. It has been reported that AGEs trigger the activation of NF-*κ*B by interaction with receptor for AGE (RAGE), leading to its translocation to the nucleus where it induces transcription, and the promoter region of the RAGE gene contains NF-*κ*B binding sites, potentially producing a self-perpetuating pathway. Moreover, the AGE-RAGE interaction activates TGF-*β*_1_ signaling pathways and subsequently induces mesangial cell hypertrophy and glomerular sclerosis by ECM synthesis [2]. Therefore, AGEs accumulation in the kidney has been regarded as an index of progressive renal damage in diabetic nephropathy. Actually, not only the overexpression of AGEs but also the higher levels of NF-*κ*B and TGF-*β*_1_ in kidney of STZ-diabetic rats were alleviated by 12 weeks of 2.8 g kg^−1^ per day DSS treatment. It seems that DSS influenced not only the AGE-RAGE signaling but also the NF-*κ*B-TGF-*β*_1_-dependent pathway to some extent, thus leading to attenuate renal damage caused by the protein glycation reaction. 

When considering AGEs from another viewpoint, CML, pentosidine, and methylglyoxal derivatives are among some of the well-characterized compounds that commonly are used as AGE markers [28]. Particularly, CML is not only referred to as a glycoxidation product, but is also formed during the metal-catalyzed oxidation of polyunsaturated fatty acids in the presence of protein [29]. Therefore, CML could serve as a general biomarker of oxidative stress resulting from carbohydrate and lipid oxidation reactions. We found that treatment of diabetes with 12 weeks of DSS not only lowered the renal CML level but also decreased the accumulation of lipid peroxidation products in kidney. The results indicated that the beneficial effect of DSS on diabetic nephropathy was linked to reduce the intensity of oxidative stress. 

Diabetes exhibits high oxidative stress due to persistent and chronic hyperglycemia, which thereby depletes the activity of antioxidative defense system and thus promotes de novo free radicals generation; strategies to reduce oxidative stress in diabetes mellitus may exert favorable effects on the progression of diabetic glomerulosclerosis [30, 31]. Among antioxidative enzymes, SOD catalyzes dismutation of the superoxide anion into hydrogen peroxide, while GSH-Px both detoxifies hydrogen peroxides and converts lipid hydroperoxides to nontoxic alcohols; thus, antioxidant enzymes activities could reflect antioxidant defense status [32]. In this work, the reduced activities of SOD and GSH-Px in kidney of STZ-diabetic rats were elevated by DSS. The classical formula protecting kidney of diabetic rats from oxidative damage by enhancing enzymatic antioxidative defense systems could be considerable. 

The findings of the present study are of merit in revealing, for the first time, that DSS has an antidiabetic property with plasma glucose lowering action to reduce hyperglycemia-induced generation reactive oxygen species. DSS also, with the capacity to ameliorate the defective antioxidative defense system, leads to modulate the oxidative stress, thereby resulting in downregulation of NF-*κ*B as well as TGF-*β*_1_ and consequently attenuation of type IV collagen expression in diabetic renal cortex tissue. Although DSS did not produce statistically significant effect on the amelioration of diabetic-dependent alterations in urinary albumin, albumin excretion rate, and creatinine clearance, this classical formula can prevent or retard the development of diabetic nephropathy via its beneficial effects for correcting the hyperglycemia, antioxidant enzyme system, renal dysfunction and protecting against histopathological changes in the kidneys of diabetic rats which cannot be neglected. The Chinese prescription may therefore possess utility as adjuvant therapy for control of diabetes and its complications.

Free radicals play vital role in the inflammation, dementia as well as diabetic nephropathy, and the other diseases [33]. Traditionally, DSS was used to relieve menorrhalgia and other abdominal pains of women [12, 13]. The therapeutic effect of DSS on Alzheimer's disease was also reported [14, 15]. Beneficial effect of DSS on AGEs-mediated renal injury in diabetic rats has been clarified in the present study. The potential relationships among these DSS-treated diseases might be related to the capacity of this prescription on reducing oxidative stress under diseases. 

Most TCM remedies are formulated to contain different herbs in combination in order to enhance the curative efficacy and also reduce the side effects. Monoterpene glycosides, phenolic compounds, and phthalides are the most representative components of DSS as far as both the contents and their biological activities are concerned [6–11]. Further studies will be required to identify the major active constituents in DSS that is responsible for the beneficial renal effects observed in the present study. 

In conclusion, DSS showed an antidiabetic effect via reducing hyperglycemia to attenuate AGEs expression and downregulate NF-*κ*B-TGF-*β*_1_ pathway in diabetic glomeruli, consequently lessening ECM deposition in renal tissue. Besides, the oxygen radical scavenging properties of DSS for amelioration of diabetic nephropathy cannot be excluded.

## Figures and Tables

**Table 1:** Changes of the body weights, plasma parameters, and SBP in STZ-diabetic rats receiving DSS treatment.

	Period (week)	Body weight (g)	Plasma glucose (mg dL^−1^)	Plasma cholesterol (mg dL^−1^)	Plasma triglyceride (mg dL^−1^)	SBP (mm Hg)
Nondiabetic rats						
Vehicle	0	193.3 ± 11.2	92.8 ± 2.4^d^	68.5 ± 4.1^c^	94.2 ± 7.6^d^	87.3 ± 3.1^c^
	4th	223.8 ± 10.4^c^	93.6 ± 3.4^d^	69.4 ± 3.8^c^	100.4 ± 6.8^d^	89.4 ± 2.9^c^
	8th	247.6 ± 13.1^c^	95.4 ± 2.7^d^	70.2 ± 3.4^c^	98.4 ± 8.2^d^	90.3 ± 3.7^c^
	12th	262.6 ± 12.3^c^	96.8 ± 3.1^d^	71.3 ± 3.6^c^	98.6 ± 6.4^d^	92.6 ± 3.4^c^

STZ-diabetic rats						
Vehicle	0	183.7 ± 10.5	408.6 ± 3.7^b^	110.9 ± 4.2^a^	338.4 ± 8.3^b^	115.5 ± 4.4^a^
	4th	176.2 ± 9.5^a^	412.5 ± 3.8^b^	114.6 ± 3.9^a^	345.6 ± 9.2^b^	124.6 ± 3.7^a^
	8th	168.3 ± 8.9^a^	418.7 ± 4.1^b^	118.1 ± 4.1^a^	349.2 ±12.4^b^	129.8 ± 3.9^a^
	12th	160.5 ± 11.4^a^	423.2 ± 4.4^b^	118.4 ± 4.7^a^	353.5 ± 10.1^b^	134.5 ± 3.6^a^
DSS (2.8 g kg^−1^ per day)	0	184.1 ± 9.7	409.6 ± 4.1^b^	112.3 ± 4.8^a^	339.2 ± 8.1^b^	115.1 ± 4.3^a^
	4th	180.2 ± 11.3^a^	405.7 ± 5.6^b^	112.1 ± 3.7^a^	344.2 ± 11.3^b^	122.4 ± 3.5^a^
	8th	177.4 ± 9.5^a^	390.2 ± 3.8^b, c^	116.8 ± 3.2^a^	348.5 ±10.1^b^	120.6 ± 4.1^a^
	12th	170.1 ± 10.9^a^	381.5 ± 4.2^b, c^	115.3 ± 3.9^a^	340.3 ± 8.1^b^	130.1 ± 4.5^a^

Values (mean ± SD) were obtained for each group of 8 animals. ^a^ *P* < 0.05 and ^b^ *P* < 0.01 compared to the values of vehicle-treated nondiabetic rats at the corresponding time, respectively. ^c^ *P* < 0.05 and ^d^ *P* < 0.01 compared to the values of vehicle-treated STZ-diabetic rats at the corresponding time, respectively.

**Table 2:** Changes of the renal function-related parameters in STZ-diabetic rats receiving DSS treatment.

Groups	Period (week)	Serum Cr (mg dL^−1^)	BUN (mg dL^−1^)	Urine volume (mL^−1^)	Ccr (ml min^−1^ per kg)	UAER (*μ*g 24 h^−1^)
Nondiabetic rats						
Vehicle	0	0.33 ± 0.09^c^	17.3 ± 2.6^d^	10.9 ± 2.8^d^	0.65 ± 0.15^d^	2.8 ± 0.2^d^
	4th	0.34 ± 0.12^c^	17.8 ± 3.1^d^	11.4 ± 3.2^d^	0.62 ± 0.17^d^	2.6 ± 0.4^d^
	8th	0.34 ± 0.09^c^	18.4 ± 2.8^d^	11.7 ± 2.9^d^	0.64 ± 0.14^d^	2.7 ± 0.2^d^
	12th	0.35 ± 0.06^c^	18.2 ± 3.4^d^	12.1 ± 3.5^d^	0.68 ± 0.13^d^	2.9 ± 0.3^d^

STZ-diabetic rats						
Vehicle	0	0.51 ± 0.06^a^	28.4 ± 3.3^b^	25.4 ± 3.7^b^	1.56 ± 0.21^b^	15.4 ± 2.1^b^
	4th	0.53 ± 0.08^a^	29.7 ± 2.7^b^	26.9 ± 2.5^b^	1.64 ± 0.18^b^	16.3 ± 1.9^b^
	8th	0.58 ± 0.07^a^	31.6 ± 3.6^b^	30.2 ± 2.8^b^	1.73 ± 0.15^b^	17.5 ± 2.6^b^
	12th	0.64 ± 0.08^a^	33.6 ± 2.9^b^	33.6 ± 3.2^b^	1.80 ± 0.19^b^	19.4 ± 3.2^b^
DSS (2.8 g kg^−1^ per day)	0	0.50 ± 0.06^a^	28.6 ± 2.8^b^	25.3 ± 3.2^b^	1.55 ± 0.23^b^	14.9 ± 2.6^b^
	4th	0.50 ± 0.10^a^	27.8 ± 3.2^b^	24.8 ± 3.4^b^	1.58 ± 0.16^b^	13.7 ± 2.5^b^
	8th	0.51 ± 0.12^a^	28.2 ± 3.4^b^	26.2 ± 2.9^b^	1.52 ± 0.18^b^	14.8 ± 2.2^b^
	12th	0.54 ± 0.09^a^	27.2 ± 3.5^b^	27.8 ± 2.8^b^	1.43 ± 0.21^b^	15.1 ± 3.1^b^

Values (mean ± SD) were obtained for each group of 8 animals. ^a^ *P* < 0.05 and ^b^ *P* < 0.01 compared to the values of vehicle-treated nondiabetic rats at the corresponding time, respectively. ^c^ *P* < 0.05 and ^d^ *P* < 0.01 compared to the values of vehicle-treated STZ-diabetic rats at the corresponding time, respectively.

**Table 3:** Changes of the hypertrophy-related parameters in STZ-diabetic rats receiving 12 weeks of DSS treatment.

Groups	Kidney weight (g)	Kidney weight/body weight (mg g^−1^)
Nondiabetic rats		
Vehicle	1.5 ± 0.2^d^	5.6 ± 0.4^d^
STZ-diabetic rats		
Vehicle	2.9 ± 0.3^b^	16.5 ± 0.5^b^
DSS (2.8 g kg^−1^ per day)	2.1 ± 0.3^b^	12.3 ± 0.2^b, c^

Values (mean ± SD) were obtained for each group of 7 animals. ^b^ *P* < 0.01 compared to the values of vehicle-treated nondiabetic rats. ^c^ *P* < 0.05 and ^d^ *P* < 0.01 compared to the values of vehicle-treated STZ-diabetic rats, respectively.

**Table 4:** Renal levels of AGEs, CML, lipid peroxidation products, and antioxidant enzyme activity in STZ-diabetic rats receiving 12 weeks of DSS treatment.

Groups	AGEs (AU)	CML (ng mg^−1^)	Lipid peroxidation products (nmol mg^−1^)	SOD (U mg^−1^)	GSH-Px (U mg^−1^)
Nondiabetic rats					
Vehicle	3.1 ± 0.2^d^	20.2 ± 4.3^d^	6.4 ± 0.6^d^	12.3 ± 0.8^d^	2.8 ± 0.4^c^
STZ-diabetic rats					
Vehicle	6.8 ± 0.3^b^	43.3 ± 5.6^b^	14.8 ± 1.2^b^	6.7 ± 0.6^b^	1.5 ± 0.2^a^
DSS (2.8 g kg^−1^ per day)	4.3 ± 0.2^a, c^	30.4 ± 3.7^a, c^	10.3 ± 0.7^a, c^	10.1 ± 0.5^a, c^	2.3 ± 0.2^ c^

Values (mean ± SD) were obtained for each group of 7 animals. ^a^ *P* < 0.05 and ^b^ *P* < 0.01 compared to the values of vehicle-treated nondiabetic rats, respectively. ^ c^ *P* < 0.05 and ^d^ *P* < 0.01 compared to the values of vehicle-treated STZ-diabetic rats, respectively.
